# The prevalence and socio-economic determinants of HIV among teenagers aged 15–18 years who were participating in a mobile testing population based survey in 2013–2014 in Zambia

**DOI:** 10.1186/s12889-016-3449-3

**Published:** 2016-08-15

**Authors:** Pascalina Chanda-Kapata, Eveline Klinkenberg, Nicole Maddox, William Ngosa, Nathan Kapata

**Affiliations:** 1 Department of Disease Surveillance, Control and Research, Ministry of Health, Lusaka, Zambia; 2 KNCV Tuberculosis Foundation, The Hague, Netherlands; 3 Department of Global Health, Amsterdam Institute for Global Health and Development, Academic Medical Centre, Amsterdam, The Netherlands; 4 Department of Epidemiology and Disease Control, Ministry of Community Development, Mother and Child Health, Lusaka, Zambia

**Keywords:** Teenagers, HIV/AIDS, Population survey, Zambia, Infectious diseases, Youth, Adolescents, Mobile testing

## Abstract

**Background:**

The objective of the study was to estimate the prevalence of HIV among teenagers in Zambia and determine whether age, sex, setting, educational level, marital and socioeconomic status were associated with being HIV positive.

**Methods:**

A cross sectional population based survey of the prevalence of HIV among teenagers aged 15–18 years old who were also participants in a national Tuberculosis (TB) prevalence survey. Consenting teenagers were counselled and tested for HIV. The HIV prevalence was estimated using a logistic regression model. Associations of social demographic characteristics with HIV were determined using univariate and multivariate.

**Results:**

The study involved 6,395 teenagers aged 15–18 years where 2,532 declined HIV testing, 44 tested positive and 3,806 tested negative. The HIV prevalence was estimated to be 1.1 % (95 % CI 0.71-1.60); in females the HIV prevalence was 1.6 % (95 % CI 0.99-2.20) whereas in males it was 0.58 % (95 % CI 0.10-1.10). The prevalence of HIV was twice as high among the urban (1.90 %; 95 % CI 0.99-2.90) than the rural teenagers (0.89 %; 95 % CI 0.46-1.30), and being divorced or widowed was associated with higher risk of HIV regardless of residence. The risk of HIV was lower among students or those who were in school compared to those who were unemployed and not in school.

**Conclusion:**

HIV prevalence among teenagers was lower than the overall national level prevalence. The patterns of HIV risk among the young population will require further monitoring in order to identify appropriate tools for intervention.

## Background

According to the World Health Organization (WHO) definition, adolescence is the transient phase of life for people aged 10–19 years when the process of sexual exploration begins [1]. During this period of biological and psycho-social transition experimentation increases the vulnerability of this group to contracting sexually transmitted infections, specifically HIV [2]. For decades, HIV rates among teenagers and young adults (aged 15–24) have been the fastest growing categories of the share of people living with the disease because of the increasing HIV burden within this age group. In 2013 alone, there were 670,000 new cases of HIV observed in young people between the ages of 15 to 24, and of these 250,000 (37 %) were teenagers aged 15 to 19 [3]. Over one third of people living with HIV worldwide are said to have been infected between the ages of 15 and 24 [4] and AIDS is the number one killer among adolescents in Africa [5].

Since Zambia’s first reported AIDS diagnosis in 1984, HIV prevalence among adults remained stagnant throughout the nineties at around 20 %, started to decline in 2000 reaching 16 % in 2001 [6] and continued to decrease. The prevalence rate showed a similar decline among adolescents with a level of 28 % in 1993 declining to 15 % in 1998 [7] and further down to 9 % in 2006 with a prevalence of 4 % in 2013 [8]. Currently, HIV prevalence among adults aged 15–49 stands at 13 % [8]. The rate of newly acquired HIV infections is lowest among teenagers aged 15–19 years in Zambia compared to adults.

This article presents new data on estimated prevalence of HIV among teenagers aged 15–18 years in a population based survey. Further the article assesses the association of the various social determinants with HIV. This information will provide baseline data that will be essential to investigate the progress made in reducing prevalence and to address some of the remaining challenges for teenagers with HIV in Zambia. The decision to conduct a special analysis in this age group is based on the fact that teenagers, in Zambia, are more likely to engage in unprotected sex, are likely to lose their virginity before 15, and practice sex with people ten or more years their senior [8], thereby increasing their risk of HIV infection. Evidence from elsewhere also shows that in Africa, HIV/AIDS causes significant mortality among older adolescents [4] and Dellar and colleagues [9] have emphasized that young women in sub-Saharan Africa are a key population that will urgently require attention and appropriate mitigation interventions. Additionally, Prevention of Mother to Child Transmission (PMTCT) programs from late 1990s until the early 2000s were non-existent [10] and therefore this group at present may also represent many survivors of mother-to-child transmission of HIV and therefore understanding and determining the prevalence in this group is cardinal. In addition, data on this key population, using sociodemographic factors is also limited in Zambia, and such data could be useful in assessing differentials by age and sex at national and regional levels.

## Methods

This was a cross sectional survey of teenagers aged 15–18 years old who were participating in a population based National TB Prevalence survey in Zambia. HIV consent procedures were commenced after the participants had completed the TB survey component. HIV testing and counselling were conducted only among participants who provided written informed consent and obtained assent from their parent/caretaker. Those who consented had their blood collected through a finger prick. Blood was placed directly onto the screening rapid test strip (Determine®). A unique identification number (barcode) was linked to the social and demographic variables which were already collected from each participant. No other identifiers were collected. Those who declined to participate were allowed to exit the study.

All study participants had an option of whether to know the results of the HIV test or not. Pre-test counselling was done prior to offering the test. For participants, who did not wish to know their HIV test result, the post-test counselling was not performed. The HIV test result was available within 10–20 min. If the participant wished to know the result of the test, post-test counselling was performed. Trained nurses (certified in standard national Voluntary Counselling and Testing (VCT) programs) performed the pre and post counselling. If the test was non-reactive, a negative result was communicated to the participant. However, if the test was reactive, the participant was asked to give a second blood sample from another finger prick for a confirmatory test (Uni-Gold® Recombigen®). If the second test was reactive, the participant was informed of the positive status, appropriately counselled and referred to the nearest health facility for further management. If the second test was non-reactive, the result was considered indeterminate and the participant was advised to visit the nearest health facility for a further testing after six weeks.

The data was analysed using STATA *Version* 12.0. Comparison of categorical variables was done through the chi-square tests. For prevalence estimation; individual level modelling was performed. As a first step a simple cluster level analysis was done whereby the prevalence rates were calculated at cluster level and then combined to one single point estimate with confidence boundaries. The second step was an individual level model analysis whereby logistic regression models were applied restricted to participants with complete outcomes only, so called complete case analysis. Individuals with missing data on outcome, i.e. HIV status, were excluded.

Univariate and multivariate logistic regression were performed to determine the relationship between HIV status and one or more independent variables. The variables assessed were age, sex, residency setting, education level, marital status, employment status and socioeconomic status.

The study protocol was cleared by the University of Zambia Biomedical Research Ethics Committee (UNZABREC) No: 020-08-12. Authorisation to conduct the survey was sought in line with the existing national policies and guidelines at national, provincial and district levels. The Institutional Review Board (IRB) approved this consent procedure. Written informed consent was obtained from those aged 18 years. In the case of minors aged 15–17 years, parents/legal guardians provided the consent while minors provided assent. The consent was recorded on standard forms which were developed for the study and these were filed in lockable cabinets at the end of each cluster operation.

## Results

A total of 6,395 teenagers aged 15–18 years (mean age 16.5; SD =1.1 years) participated in the prevalence survey and were used in this analysis. Table 1 outlines the background characteristics of the study population. The majority were female, from a rural area and more than half had a secondary education. About 7.4 % reported being married or living as married; 68.8 % were students and 28.7 % were from the highest wealth quintile.

**Table 1:** Consent to HIV testing by age, sex, setting and wealth quintile

Variable	Total	Agreed to HIV test (*N* =3863) (%)	Declined HIV test (*N* = 2532) (%)
Age (*N* = 6395)			
15	1657	867 (24.4)	790 (31.2)
16	1615	938 (24.3)	677 (26.7)
17	1564	938 (24.3)	626 (24.7)
18	1559	1120 (29.0)	439 (17.3)
Sex (*N* = 6395)			
Male	3067	1736 (44.9)	1321 (52.2)
Female	3338	2127 (55.1)	1211 (47.8)
Setting (*N* = 6395)			
Rural	4220	2935 (76.0)	1285 (50.8)
Urban	2175	928 (24.0)	1247 (49.2)
Wealth quintile (*N* = 5456)			
Lowest	771	550 (16.9)	221 (10.1)
Second lowest	946	650 (20.0)	296 (13.5)
Middle	1077	711 (21.8)	366 (16.7)
Fourth	1095	616 (18.9)	479 (21.8)
Highest	1567	736 (22.6)	831 (37.9)

Of the 6,395 teenagers included, about 40 % declined HIV testing (Table 1). The proportion declining to test was highest among young men, 15 year olds, rural residents, and individuals from the highest wealth quintile. Of those who tested, almost everyone wanted to know their test results.

The overall test results are summarised in Table 2. Forty-four were found to be HIV seropositive with 13 being indeterminate.

**Table 2:** Overall HIV test results

Variable	Frequency	Proportion (%)	95 % CI
Positive	44	1.1	0.8-1.5
Negative	3806	98.5	98.1-98.9
Indeterminate	13	0.3	0.2-0.6
Total	3863	100.0	

The estimated crude and cluster level prevalence of HIV was found to be 1.1 % and 1.4 % respectively. The estimated individual level prevalence of HIV was found to be 1.1 %. The outcomes of the three methods were similar and therefore we take the best model as final, which is model three.

The prevalence of HIV varied significantly by age, sex, setting, education level, wealth quintile, marital status and occupation as shown in Table 3. By age, the highest prevalence was found among the 18 year olds at 1.5 %. The prevalence of HIV in the female participants was more than twice that of males and the prevalence among urban residents was also twice that of their rural counterparts. The prevalence for HIV was similar among those who had some primary and secondary education and in those who had a tertiary education. Individuals in the lowest quintile were found to have the lowest HIV prevalence compared to those in other quintiles. The teenagers who were divorced had the highest HIV prevalence in relation to the other marital status categories and the prevalence in married teenagers was less than half. The unemployed teenagers had the highest HIV prevalence while the pupils reported the least.

**Table 3:** Estimation of HIV Prevalence among 15–18 year old by background characteristics

Variable	Prevalence N (%)	95 % Binomial Confidence interval
Individual level model	44 (1.1)	0.7-1.6
Age		
15	8 (0.9)	0.4-1.8
16	7 (0.8)	0.3-1.5
17	12 (1.3)	0.7-2.2
18	17 (1.5)	0.9-2.4
Sex		
Male	10 (0.6)	0.1-1.1
Female	34 (1.6)	1.1-2.2
Setting		
Rural	26 (0.9)	0.5-1.3
Urban	18 (1.9)	1.0-2.9
Education Level (*N* = 6,395)		
No schooling	2 (3.6)	0.0-10.6
Primary	15 (1.0)	0.46-1.6
Secondary	26 (1.1)	0.62-1.6
Tertiary	1 (6.7)	0.0-19.5
Wealth Quintile		
Lowest	1 (0.2)	0.0-1.0
Second lowest	7 (1.1)	0.4-2.2
Middle	13 (1.8)	1.0-3.1
Fourth	7 (1.1)	0.5-2.3
Highest	10 (1.4)	0.7-2.5
Marital status		
Never married	36 (1.0)	0.7-1.4
Currently married	7 (2.0)	0.8-4.0
Divorced	1 (5.9)	0.1-28.7
Widowed	0 (0.0)	-
Occupation		
Unemployed (1277)	24 (1.9)	1.2-2.8
Employed (117)	2 (1.7)	0.2-6.0
Pupil/student (2469)	18 (0.7)	0.4-1.1

Univariable and multivariable analysis showed that the teenage males were three times less likely to be HIV positive than females as shown in Table 4. Urban teenagers were 2.2 times more likely to be HIV positive than rural teenagers. Students were associated with a three times lower chance of having HIV than the unemployed.

**Table 4:** Univariable and multivariable analysis of HIV with participant background characteristics

	Univariable		Multivariable	
Variable	OR [95 % CI]	*p*-value	OR [95 % CI]	*p*-value
Age (years)				
15	Ref			
16	1.1 (0.5-2.6)	0.808	0.81 (0.29-2.24)	0.685
17	1.5 (0.7-3.3)	0.328	1.39 (0.57-3.43)	0.469
18	1.5 (0.7-3.2)	0.320	1.65 (0.71-3.85)	0.243
Sex				
Female	Ref			
Male	0.6 (0.3-1.0)	0.044	0.35 (0.18-0.73)	0.004
Setting				
Rural	Ref			
Urban	1.7 (1.1-3.2)	0.025	2.21 (1.21-4.05)	0.010
Education Level				
No schooling	Ref			
Primary	0.4 (0.1-1.9)	0.264	0.28 (0.06-1.26)	0.098
Secondary	0.4 (0.1-1.6)	0.175	0.31 (0.07-1.32)	0.113
Tertiary	1.9 (0.2-22.8)	0.602	1.93 (0.16-22.83)	0.603
Marital status				
Never married	Ref			
Currently or living as married	1.4 (0.6-3.1)	0.407	1.91 (0.84-4.32)	0.121
Divorced or separated	4.4 (0.6-33.7)	0.156	6.0 (0.77-46.21)	0.087
Employment status				
Unemployed	Ref			
Employed	1.5 (0.4-6.8)	0.564	1.0 (0.13-7.56)	0.989
Pupil/Student	0.4 (0.2-0.6)	0.000	0.33 (0.17-0.63)	0.001
Wealth Quintile				
Lowest	Ref			
Second lowest	1.7 (0.6-5.0)	0.334	1.8 (0.3-3.9)	0.095
Middle	2.3 (0.8-6.5)	0.100	2.3 (0.3-4.4)	0.026
Fourth	2.0 (0.7-5.7)	0.207	1.8 (0.3-3.9)	0.085
Highest	1.5 (0.5-4.4)	0.461	2.0 (0.0-4.1)	0.055

Disaggregating marital status by gender showed that married females were 1.3 times more likely to be HIV positive than their never married counterparts. Married males were 3.4 times more likely to be HIV positive than their never married counterparts.

## Discussion

The prevalence of HIV among teenagers in the age group of 15 to 18 years in Zambia according to this study was found to be 1.1 % which was much lower than what has been reported before in the similar age group and the general population figures of 13 % reported in the Zambia Demographic Health Surveys (ZDHS) in 2014 [8].

This study was conducted as part of the national TB prevalence survey and therefore the methods employed were different from those that are usually used when conducting the ZDHS. However, the fact that it was conducted in a population based survey in which all eligible participants were requested to participate in the HIV survey implies that these results can be expected to be representative of the 15 to 18 year olds in the general population. The findings therefore contribute to the body of evidence on adolescent sub-populations. Notable was also the fact that the prevalence varied significantly among the various population categories, for instance, the older teenagers aged 18 years had the highest prevalence rates among their peers. This could be because teenagers at this age may be engaging in more risky behaviours than their younger peers [11–13]. Furthermore, the burden of HIV among teenagers was found to be higher among females than males. The prevalence in females was actually more than twice that found in the males. Males were three times less likely to be HIV positive than the females.

This was consistent with what has been observed before in other studies [13–16].

Teenagers from the urban areas had a higher HIV burden than their rural counterparts with the prevalence for the urban residents being twice as much as that for the rural residents. Other studies have also shown that the HIV prevalence is higher in urban residence than in the rural residence [15, 17–19]. In South Africa, Shisana et al. [19] found that HIV prevalence was 17.6 % in urban areas in comparison to 11.6 % in the rural sectors. Another study in Zimbabwe with 200 girls showed that urban residents were more likely to have been infected with HIV than their rural counterparts [17].

Teenagers residing in urban areas were twice as likely to be HIV positive as those living in rural areas. This rural–urban divide could be attributed to HIV-risk associated behaviours. In Zambia, the female youth aged 15–19 years and living in urban areas are more likely to engage in a sexual relationship with a man 10 years older than them, have more than two sex partners, and engage in sexual intercourse while intoxicated [8, 12]. This may explain the increased risk of HIV observed in the urban adolescents. Similarly a study in Zimbabwe involving 200 girls aged 15–19, showed that girls in urban areas where more likely to be sexually active, to have been pregnant, and to have the HIV virus [17], thereby underscoring the reasons why urban teenagers may have an increased HIV risk.

Differences in educational attainment showed no significant impact on HIV status, however, high educational level was a risk factor for HIV infection. Those in tertiary school reflected the highest HIV prevalence rates compared to those in secondary and primary schools. Although schools have traditionally been used for reaching adolescents about reproductive health and HIV prevention, studies show that education does not indicate awareness or safer practices. Doyle et al. [12] found that among young people living in communities with greater access to both education and employment opportunities, there was a higher degree of risk-taking behaviours that are associated with HIV. A cross-sectional study involving 11 African countries, including Zambia, found that better educated females were more likely to have multiple partners than those with less education [12]. The same study also observed an association between the age of sex partners and education, where highly educated females (aged 15–19) living in urban areas were more likely to engage in sex with a partner who was 10 or more years older in comparison to females living in rural areas and those with lower education levels.

The prevalence of HIV was also much higher in teenagers who were divorced than in those who were currently married and those who had never been married before; however, those married also had higher HIV prevalence than those who had never been married before. The divorced or widowed teenagers had a higher risk of having HIV than those who were never married. This finding negates the presumption that marriage may offer protection against HIV since sex becomes limited to one partner [17]. Despite the common view of marriage as security, there appears to be a vulnerability factor for females. Perhaps, this protection seems only applicable to wives as the teenage married males were found to be three times more likely to have HIV than their counterpart married females. Findings from cities in Cameroon, Kenya, and Zambia show that married teenage women are more likely to be HIV-positive than unmarried women [14, 20, 21]. A cross-sectional study comparing 14 African countries found that married female adolescents were less likely than sexually experienced unmarried female adolescents to know one or more ways of avoiding HIV [12]. Even when compared with all unmarried adolescents, many of whom were sexually inexperienced, married adolescents were often poorly informed [14]. More strategies and interventions are needed to address the unique social characteristics required for safe practices throughout marriage in order to minimize the risk of HIV infections. There is also a need to investigate reasons why the risk is higher among the teenage married males in order to better understand their behavioural patterns.

Coincidentally, high income was also associated with increased risk and the risk of HIV decreased with decreasing wealth status. Interesting to note was the fact that, the teenagers who belonged to the lowest wealth quintiles had the lowest prevalence rates in relation to those in the higher wealth quintiles. The reason for this discrepancy may require further exploration through other studies.

This study has also contributed to increasing the number of youths who know their HIV status, since youth often face more challenges than adults in accessing testing services [8]. The extent of undiagnosed HIV among adolescents in Zambia is unknown and this gap poses a risk of transmitting HIV.

While there has been remarkable progress in Zambia, the data argues for further research to assess the barriers to testing and treatment, for the development of innovative testing approaches specific to this age group, in addition to expanding support services for adolescents living with HIV. Understanding these issues starts with adequate documentation for that specific age group avoiding aggregating findings with other populations. The comparison of youth between the ages of aged 15–18 on HIV indicators provides an important cornerstone for reducing HIV risk and vulnerability in Zambia. Combining this age set with older youth (>18) and young adults aged 20–24, as done in the Zambia Demographic Health Survey, makes the dynamics to be lost as key risk indicators are different within these groups. Thus, it is important to segregate these groups to understand which key factors are drivers in the epidemic affecting teenagers, and to see these variances across sociodemographic factors.

As it stands, youth constitute 1.2 billion of the world’s population and are expected to make up about 40 % of sub-Saharan Africa by 2050 [3]. It is hopeful that with data improvements and targeted interventions, HIV prevalence rates among teenagers will continue to decline based on programs that reduce risk, vulnerability, morbidity, and mortality.

This study offers a glimpse of the HIV situation in Zambia among teenagers aged 15–18 years. The data presented does not reflect programmatic effectiveness based on interventions or deal with interventions designed to mitigate the issues discussed, as this was not the scope of the study. The findings should have extended to include all teenagers starting at age 13 and ending at 19, but the design was to focus on the age that was not expected to be having sex legally and neither are they expected to be married. This is a potentially neglected group harbouring significant burden which if left unchecked can be a cause for higher morbidity and mortality from HIV at a stage when they should be progressing into economically productive adults. Such a scenario may rob the country of a productive age group with negative consequences to both the family and nation at large. The findings provide new evidence and suggest areas for further research and analysis.

## Conclusion

Understanding the burden and risk of HIV among the youth is essential for developing programmes that may help curtail the spread of HIV in this sub group. Interventions aimed at improving testing and risk perception are required. Studies aimed at understanding risky behaviours; risk perception and knowledge of HIV may be prerequisites for HIV testing or encouraging screening habits for those who become sexually active.
